# Influence of meteorological factors on the level and characteristics of culturable bacteria in the air in Gliwice, Upper Silesia (Poland)

**DOI:** 10.1007/s10453-018-9510-1

**Published:** 2018-03-01

**Authors:** Ewa Brągoszewska, Józef S. Pastuszka

**Affiliations:** Department of Air Protection, Silesian University of Technology, 22B Konarskiego St., 44-100 Gliwice, Poland

**Keywords:** Culturable bacteria, Size distribution, Bacteria identification, Outdoor air

## Abstract

Numerous studies have focused on occupational and indoor environments because people spend more than 90% of their time in them. Nevertheless, air is the main source of bacteria in indoors, and outdoor exposure is also crucial. Worldwide studies have indicated that bacterial concentrations vary among different types of outdoor environments, with considerable seasonal variations as well. Conducting comprehensive monitoring of atmospheric aerosol concentrations is very important not only for environmental management but also for the assessment of the health impacts of air pollution. To our knowledge, this is the first study to present outdoor and seasonal changes of bioaerosol data regarding an urban area of Poland. This study aimed to characterize culturable bacteria populations present in outdoor air in Gliwice, Upper Silesia Region, Poland, over the course of four seasons (spring, summer, autumn and winter) through quantification and identification procedures. In this study, the samples of bioaerosol were collected using a six-stage Andersen cascade impactor (with aerodynamic cut-off diameters of 7.0, 4.7, 3.3, 2.1, 1.1 and 0.65 μm). Results showed that the concentration of airborne bacteria ranged from 4 CFU m^−3^, measured on one winter day, to a maximum equal to 669 CFU m^−3^ on a spring day. The average size of culturable bacterial aerosol over the study period was 199 CFU m^−3^. The maximal seasonally averaged concentration was found in the spring season and reached 306 CFU m^−3^, and the minimal seasonally averaged concentration was found in the winter 49 CFU m^−3^. The most prevalent bacteria found outdoors were gram-positive rods that form endospores. Statistically, the most important meteorological factors related to the viability of airborne bacteria were temperature and UV radiation. These results may contribute to the promotion and implementation of preventative public health programmes and the formulation of recommendations aimed at providing healthier outdoor environments.

## Introduction

Conducting comprehensive monitoring of atmospheric aerosol concentrations is very important not only for environmental management but also for the assessment of the health impacts of air pollution. An aerosol may be defined as a relatively time-stable two-phase system consisting of finely divided particles (that can be solid or liquids) suspended in a gas (which is air). A special type of aerosol is bioaerosols, being a two-phase system consisting of airborne biological particles. Biological particles suspended in the air may consist of bacteria, fungi, viruses and/or pollen, fragments of the above or their metabolic products (e.g. mycotoxins) and endotoxins (part of the outer membrane of the cell wall of gram-negative bacteria). Such particles may be suspended in the air either as individual organisms or attached to dust particles or tiny droplets of water (Adhikari et al. 2006; Lighthart 1997).

The smallest bioparticles are viruses, the size of which varies from 0.02 µm to 0.3 µm. Bacteria and microscopic fungi cells are 0.3 to 100 µm and pollen, and other biological particles range from 10 µm up to even hundreds of micrometres (Haas et al. 2010; Kołwzan et al. 2005; Libudzisz et al. 2008; Nevalainen et al. 1993).

Bioaerosols can be the cause of a variety of infectious diseases as well as cause allergic and toxic reactions (Arancibia et al. 2002; Liebers et al. 2008; Thorn and Kerekes 2001). Infectious and non-infectious diseases caused by inhalation of different bioaerosols depend not only on the biological properties and chemical composition of these bioaerosols but also on the inhaled quantity and the site of their deposition in the respiratory tract (Degobbi et al. 2011). Because the deposition site in the respiratory tract is directly related to the aerodynamic diameter of the particles, the health effect depends highly on their physical properties, and especially on their size distribution (Nasir and Colbeck 2010; Nevalainen et al. 1993).

Bioaerosols are to a great extent of natural origin (e.g. rotting leaves, mould growth in damp areas) and are therefore ubiquitously present in the environment (Nasir and Colbeck 2010). It has been reported that approximately 24% of the total atmospheric particles and 5–10% of the total suspended particulate mass were contributed by bioaerosol (Walser et al. 2015). Spracklen and Heald (2014) found that fungal spores and bacteria contributed 8% and 5%, respectively, to global continental mean supermicron number concentrations.

Numerous studies have focused on occupational and indoor environments (Brągoszewska et al. 2016; Dumała and Dudzińska 2013; Mainka et al. 2015; Mentese et al. 2012; Moon et al. 2014; Pastuszka et al. 2000, 2005; Ramachandran et al. 2005; Salleh et al. 2011; Stryjakowska-Sekulska et al. 2007) because people spend more than 90% of their time indoors (Ashmore and Dimitroulopoulou 2009; Lee and Chang 2000; Wichmann et al. 2010). Nevertheless, atmospheric air is the source of bacteria in indoor air and outdoor exposure is also crucial. Worldwide studies have indicated that bacterial concentrations vary among different types of outdoor environments, with considerable seasonal variations as well (Haas et al. 2010; Jeon et al. 2011; Woo et al. 2013).

The aim of this study was to investigate the concentration levels of culturable bacterial aerosols, size distributions and seasonal (spring, summer, autumn and winter) variations in outdoor air in Gliwice, a town in southern Poland. We focused on culturable bacteria only because these micro-organisms are very sensitive to interaction with other air pollutants and seem to be highly influenced by various meteorological factors, including air temperature, relative humidity (RH) and wind speed, as well as UV radiation. Therefore, we tried to find (a) the concentration levels of culturable airborne bacteria during each of the four seasons, (b) the size distributions of those bacteria and (c) the difference between bacterial community structures in different seasons.

To our knowledge, this is the first study to present outdoor and seasonal changes of bioaerosol data regarding an urban area of Poland. There are currently no formally regulated standards for bioaerosol levels in Poland. This study can thus increase awareness and provide references for a better understanding of outdoor air quality (OAQ) in the urban areas of developing countries.

## Materials and methods

**Fig. 1:**
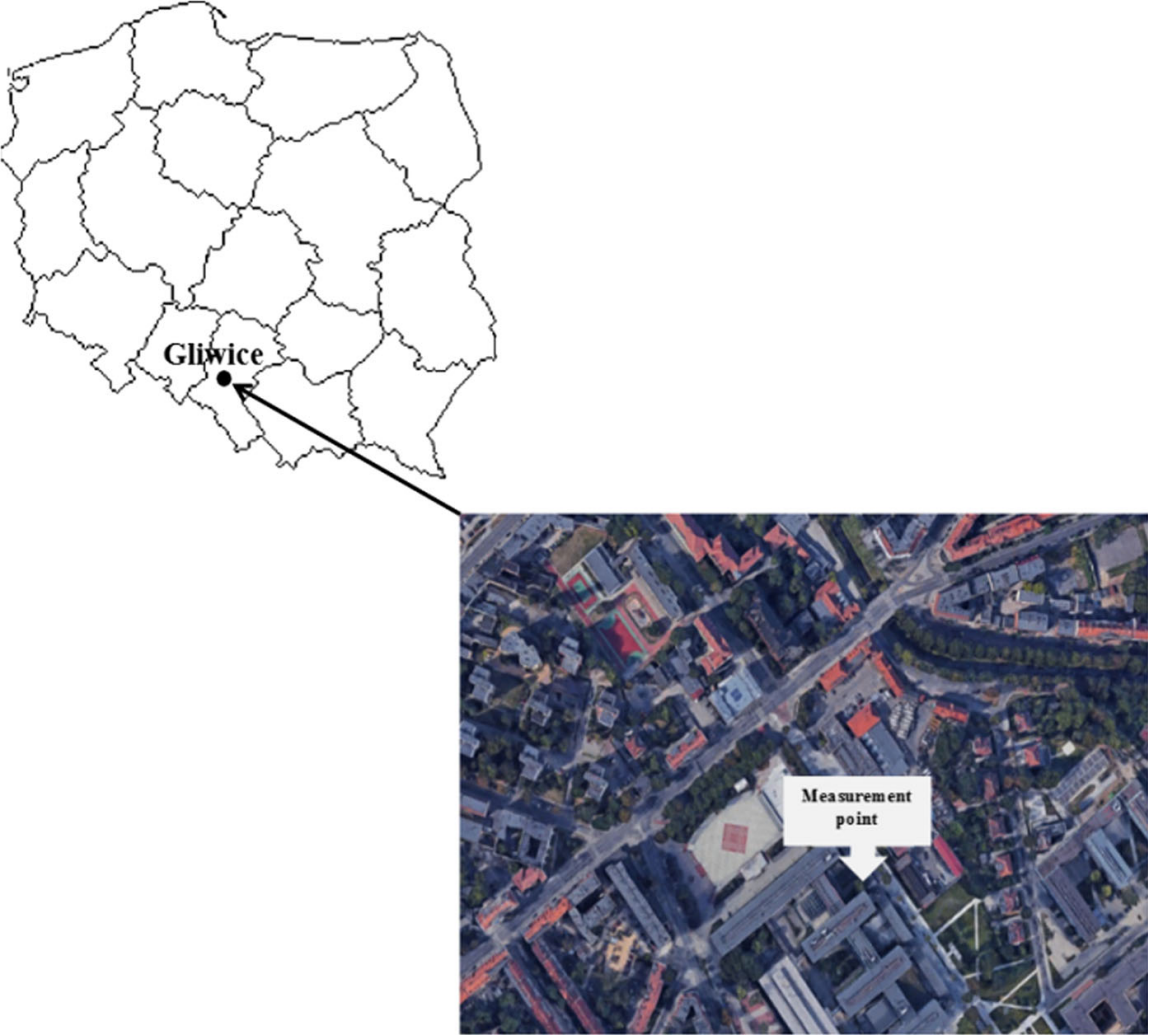
Localization of the measurement point in Gliwice (Map data: 2016© Google, ORION-ME)

The study was carried out in Gliwice (50°17′37.1″N 18°40′54.9″E) (Fig. 1). Gliwice is a typical city in the industrial region of Upper Silesia, Poland, with 4.5 million people in the region. The measurement point was located in the city centre, about 500 m from a busy road. The nearest surroundings of the measurement point are characterized by compact building development. Buildings, roads, pavements, etc., cover most of the surfaces in this part of the city.

Air sampling was conducted in four seasons (spring, summer, autumn and winter) from February 2011 to December 2012. For the spring season, the conventionally assumed months are from 21st March to 21st June, for the summer months from 22nd June to 22nd September, for the autumn months from 23rd September to 21st December and for the winter months from the 22nd December to 20th March.

During the measurements, meteorological parameters were recorded using the weather station *Oregon Scientific WMR200* (Tables 1, 2).

**Table 1:** Meteorological parameters during sample collection in each season

	Outdoor parameters (mean ± SD)			
	Spring	Summer	Autumn	Winter
	Mar 21–Jun 21	Jun 22–Sep 22	Sep 23–Dec 21	Dec 22–Mar 20
Temperature (°C)	18.7 ± 4.9	25.6 ± 4.9	7.7 ± 6.3	−4.7 ± 3.5
Relative humidity (RH) (%)	55.0 ± 19.2	53.0 ± 11.5	66.1 ± 17.0	65.0 ± 18.0
Wind velocity (km/h)	11.0 ± 4.6	13.1 ± 3.7	14.0 ± 6.0	15.2 ± 6.0
Atmospheric pressure (hPa)	1002.0 ± 7.0	1006.0 ± 6.0	1004.7 ± 9.0	1004.1 ± 8.9
UV radiation (W/m^2^)	506.0 ± 263.0	566.7 ± 189.6	172.4 ± 139.0	143.65 ± 118.7

*SD* standard deviation

**Table 2:** Descriptive statistics of meteorological conditions in Gliwice by month during sample collection (mean ± SD)

	Temperature (°C)	Relative humidity (RH) (%)	Wind velocity (km/h)	Atmospheric pressure (hPa)	UV radiation (W/m^2^)
Jan	−4.6 ± 2.8	77.0 ± 11.7	18.9 ± 2.4	1006.0 ± 8.7	157.2 ± 137.6
Feb	−5.3 ± 3.6	77.8 ± 10.4	13.2 ± 5.2	1006.0 ± 8.0	150.1 ± 104.3
Mar	−0.5 ± 0.57	76.0 ± 14.7	10.0 ± 11.5	989.0 ± 10.4	236.5 ± 212.5
Apr	17.7 ± 6.4	76.8 ± 15.4	8.0 ± 4.3	1003.7 ± 4.0	406.1 ± 299.0
May	17.1 ± 4.3	76.9 ± 13.6	11.0 ± 4.0	1000.3 ± 9.7	468.1 ± 266.0
Jun	20.2 ± 2.8	70.6 ± 9.0	13.6 ± 4.0	1002.6 ± 5.3	548.4 ± 219.0
Jul	27.5 ± 3.3	74.3 ± 9.3	11.7 ± 3.4	1008.2 ± 6.8	585.9 ± 229.7
Aug	28.2 ± 3.4	68.7 ± 5.1	13.4 ± 4.3	1005.1 ± 7.4	641.7 ± 125.2
Sep	20.6 ± 4.0	75.7 ± 6.7	14.5 ± 4.5	1002.5 ± 7.0	440.1 ± 160.5
Oct	11.1 ± 2.0	84.6 ± 6.8	11.8 ± 4.0	1005.0 ± 9.4	233.7 ± 136.0
Nov	6.0 ± 4.1	81.0 ± 7.6	14.0 ± 6.4	1007.0 ± 10.3	143.3 ± 76.6
Dec	1.0 ± 3.2	85.7 ± 5.2	15.2 ± 5.4	1002.6 ± 6.4	76.0 ± 44.0

*SD* standard deviation

The statistical analysis of meteorological parameters in outdoor air points to significant differences for all meteorological factors (except atmospheric pressure) between spring–autumn, spring–winter and summer–winter. For meteorological conditions, we found significant statistical differences between July, August, November, December (except atmospheric pressure and wind velocity) and the rest of the months. The difference was also statistically different (except atmospheric pressure and relative humidity) between January, February and the other months (*p* < 0.05).

### Sampling and analytical methods

Air samples were collected with a six-stage Andersen cascade impactor (Tisch Environmental, USA). The aerodynamic diameter ranges for each stage are > 7 µm (stage one), 4.7–7 µm (stage two), 3.3–4.7 µm (stage three), 2.1–3.3 µm (stage four), 1.1–2.1 µm (stage five) and 0.65–1.1 µm (stage six). The impactor is connected with a pump providing a constant flow rate (28.3 L min^−1^) during measurement. Sampling time, following Nevalainen et al. (1992, 1993), was 10 min. The bacterial aerosol was collected at the height of 1.2 m above the ground level. Micro-organisms were collected on nutrient media in Petri dishes located on all impactor stages. Impactors were disinfected by 70% ethanol-immersed cotton balls between each sampling.

Tryptic soy agar (TSA) was used, with cycloheximide added to inhibit fungal growth. Enumeration of bacteria was conducted according to the Polish Standard (‘PN-89 Z-04111/02. Air purity protection. Microbiological testing. Determination number of bacteria in the atmospheric air (imision) with sampling by aspiration and sedimentation method’ 1989). It is important to note that although direct measurement of the concentration of living airborne bacteria is extremely difficult, the commonly used substitute for the concentration of living micro-organisms present in the air is the number of *Colony-Forming Units* in the volume of air CFU m^−3^. Samples were incubated for 3–4 days at room temperature (20 °C).

Microscopic analysis of collected bacteria was based on gram strain preparations and provided data on cell size and shape, orientation to each cell and the appearance of spores. The possibility to cultivate the strains isolated on agar medium with blood addition (trypticase soy agar with 5% of the sheep blood) was also estimated. Bacteria were characterized in terms of their metabolic characteristics using the biochemical tests API (*Analytical Profile Index*). Their analysis was supported using the application APIweb (bioMérieux, Marcy-l’Etoile, France). Bacteria were grouped as gram-positive cocci, gram-positive rods that form endospores, non-sporing gram-positive rods, *actinomycetes* and gram-negative bacteria, according to their microscopic morphology.

The sampling was performed two to three times each week, per 2 years. Samples were collected between 11:00 and 12:00. Each sample included six impaction stages with Petri dishes, so in sum there were 1380 Petri dishes (without blanks) with biological material analysed during the study. Per year we collected 115 samples (28–29 samples for each season).

A particle counter (Aero Track, USA) was used to measure the amount of particulate matter contained in the air volume. This counter was set for the quantitative measurement of the concentration of particles at the same time in six ranges: 7.0, 4.7, 3.3, 2.1, 1.1 and 0.65 μm. Particle counter measuring time was about 3 min.

Statistical analyses were performed using the statistical package Statistica 12 (StatSoft). The concentration values reported below were presented as the mean values and standard deviation. Given the fact that the data were not normally disturbed (analysed with the Shapiro–Wilk test), a nonparametric method was employed. The Mann–Whitney *U* test was applied to assess seasonal differences at the sampling site and to assess monthly differences between the two most isolated groups of bacteria. Spearman’s rank correlation coefficient was used to evaluate the statistical dependence between airborne bacterial concentrations and meteorological factors, including temperature, relative humidity, wind velocity and UV radiation, separately for every season. Only between data of coarse fraction of bacteria and suspended dust were normally disturbed; therefore, Pearson correlation coefficient was examined. Between data of seasons/months and meteorological parameters, the post hoc test was used. Significant differences were determined when a probability lower than 0.05 was considered.

### Quality control

Quality control was practiced throughout the analyses to avoid any interference and minimize the risk of error. The bioaerosol analyses were continuously performed on the basis of the PN-EN 12322 standard (2005), which recommends an adequate number of culture media from each series in order to test the microbial contamination (PN-EN 12322 2005). Sterility was ensured by incubating the culture medium at a temperature appropriate for the method used for at least 3 days or > 72 h. The standard PN-EN 12322 does not specify how often the culture media must be controlled for sterility nor does it specify the temperature at which to incubate them. Therefore, the sterility testing was based on another standard (ISO 11133 2014). The testing of blank plates was performed per batch of the sample at the temperature used during the performed procedure. The blanks were not contaminated. The sampling equipment (Andersen Impactor) and laboratory equipment (laminar flow cabinet, autoclave, incubators and microscope) are regularly checked and have current certificates.

## Results and discussion

### Total concentration of bacterial aerosol

Table 3 presents the average concentrations of bacterial aerosol collected in the outdoor air in Gliwice during the analysed spring, summer, autumn and winter seasons. The first observation to be made is that the average concentration of the total bacterial aerosol collected in the outdoor air over the four seasons differed significantly. The average levels of bacterial aerosol concentrations in the outdoor air ranged from approximately 50 to 300 CFU m^−3^. The Mann–Whitney U test results showed that there was a significant difference between seasons (*p* < 0.05) such that only the difference between levels of bacterial aerosols collected summer versus autumn was statistically non-significant (*p* > 0.05).

**Table 3:** Average concentration *C* (CFU m^−3^) of total culturable bacteria in the studied outdoor air

Season	Mean	Median	Minimum	Maximum	SD
Spring	306	287	80	669	146
Summer	196	179	54	449	88
Autumn	246	263	29	529	132
Winter	49	45	4	131	34

The number of collected samples for spring was 54, for summer 58, for autumn 60 and for winter 58

*SD* standard deviation

Previously conducted studies have shown that the size of the concentration of bioaerosols is different depending on the prevailing season and climatic conditions. Geography and climate play an important role in determining the outdoor air microbial concentration because the transport of bioaerosol is primarily governed by hydrodynamic and kinetic factors, while their fate is dependent on their specific chemical composition and the meteorological factors to which they are exposed (Mouli et al. 2005). In Turkey, the highest level of airborne bacteria outdoors was found during the autumn (Aydogdu et al. 2010). Fang et al. (2007) also reported that bacterial counts were the highest in the autumn season. Some studies (Di Giorgio et al. 1996; Grzyb and Frączek 2013; Mahdy and El-Sehrawi 1997) have reported that the highest levels of airborne bacteria were recorded during the summer. Bowers et al. (2012) reported that in Colorado the maximum average concentration of bacterial aerosol in the outdoor air was in the spring season. The values obtained in Montreal showed that the annual bacterial distribution had minima in summer and winter, with maxima in spring and autumn (Lighthart and Stetzenbach 1994). Thus, the level of bacteria in the outdoor air has been shown to vary with geographical region.

In general, the lowest bacteria concentration was observed in winter. In winter, extreme conditions such as decreases in temperature and the heaviest rainfall and snowfall of the year might contribute to the decrease in bacteria levels. The studies conducted by Aydogdu et al. (2010) in Turkey reported that the lowest bacterial counts of bacterial aerosol were found in January and December, with 30 CFU and 48 CFU, respectively. The values obtained in outdoor air in Gliwice in the winter season are also comparable to the data obtained in the studies conducted in Warsaw, Poland, where the concentration of airborne bacteria in winter was 39 CFU m^−3^ (range 35–126 CFU m^−3^) (Gołofit-Szymczak and Górny 2010). Mentese et al. (2009) in Ankara, Turkey, reported that the average concentration of bacterial aerosol from December to March 2007 was 57 CFU m^−3^.

Analysing the results of bacterial aerosol concentrations occurring in the atmospheric air in Gliwice, it can be concluded that in this city the atmospheric air is relatively clean. A similar study conducted in Poznań, Poland, in 2002–2003 showed that during the summer the concentration of mesophilic bacteria reached a value of 13,000 CFU m^−3^ (Bugajny et al. 2005). In studies conducted in Beijing from June 2003 to May 2004, with high levels of traffic and human activity, concentrations of bacterial aerosol of almost 22,000 CFU m^−3^ were observed (Fang et al. 2008).

In Poland and in many other countries, legislation governing microbiological standards for air pollution has not been developed and implemented. One of the main reasons for this is the huge variety of air microflora and a large variety of methods for measuring them. Since appropriate standards and guidelines do not yet exist, expertise must be sought and research must be conducted to determine contaminant concentrations and exposures that are acceptable. Górny (2010), in a review of propositions for outdoor air, proposes a total concentration of bacteria of 5000 CFU m^−3^ as the upper limit for regulatory levels of bacterial aerosols. It can be seen that the concentration levels of airborne bioaerosols obtained in our study are below these proposed standards. Research by the Occupational Health and Safety Research Institute Robert Sauvé (IRSST) indicates that, in the case of total airborne bacteria concentrations above 1000 CFU m^−3^, possible microbial contamination justifies further investigation of the situation and potential requirements for action (Goyer et al. 2001).

### Size distribution of bacterial aerosols

**Fig. 2:**
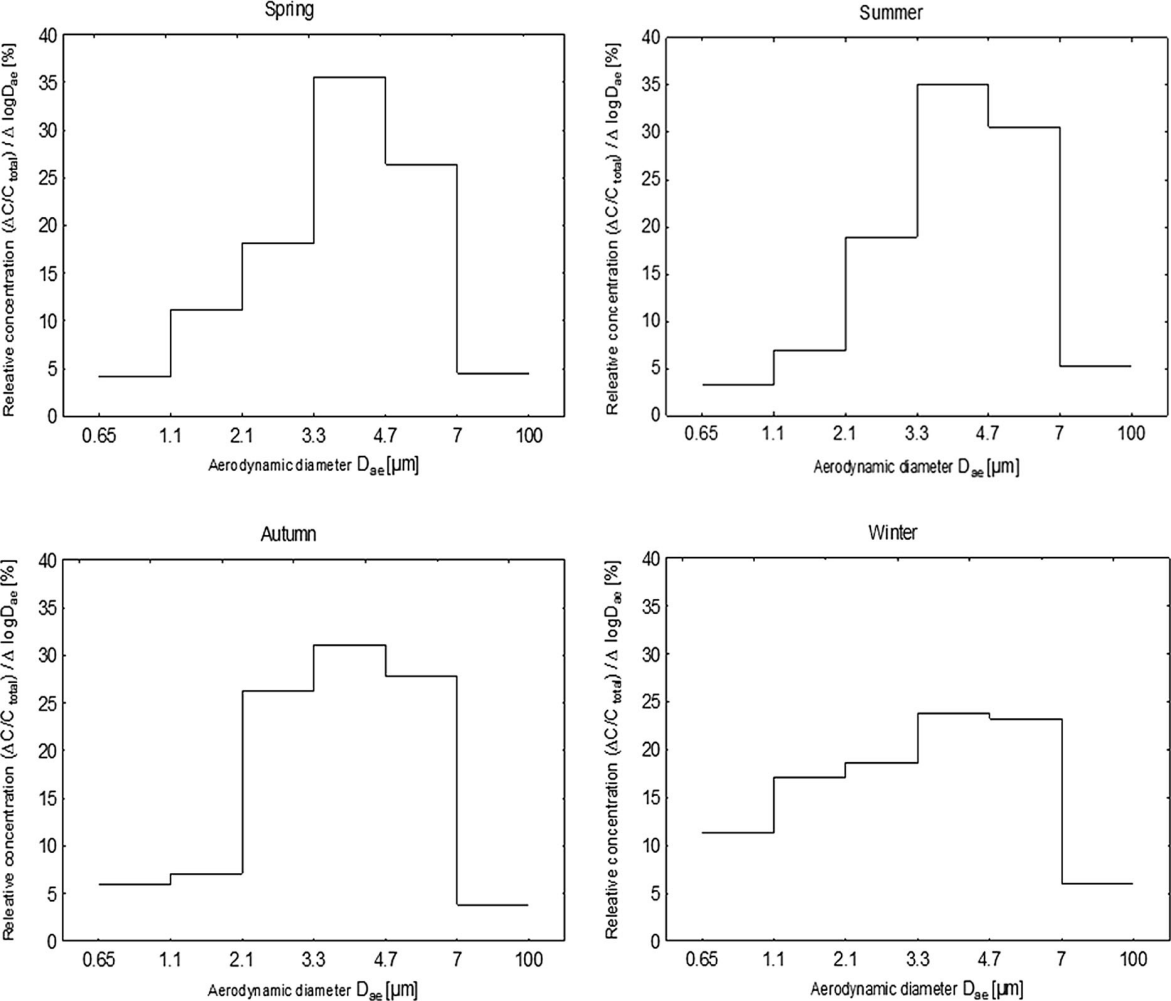
Size distribution of the bacterial aerosol in the outdoor air in the four seasons

Figure 2 shows the averaged size distributions of bacterial aerosol in the atmospheric air in different seasons. The size distributions obtained in four seasons were unimodal, with a peak falling in the range of particle bacterial aerodynamic diameters in the range from 3.3 to 4.7 µm. The peak was highest in the spring and summer (more than 35%) and lowest in winter (about 25%).

Generally, the statistical analysis of bacterial size distribution in outdoor air points to significant differences between all seasons (*p* < 0.05). However, in few cases we found that the average size distribution of bacterial aerosols was similar. The difference in size distribution was not statistically different (*p* > 0.05) at stages two and six between spring and summer and at stages two, three and six between spring and autumn. The similar size distribution of bacterial aerosol we found also at all stages of impaction (*p*_1_ = 0.43; *p*_2_ = 0.62; *p*_3_ = 0.50; *p*_4_ = 0.31; *p*_5_ = 0.94; *p*_6_ = 0.96), between summer and autumn.

In winter, particularly, the shape of the particle size distribution is rather “flattened” in a manner that cannot be easily explained. This result, however, may suggest that the sampling point is influenced by many more different sources of bacteria in spring and summer than in winter. A second reason might be that there are different profiles of bacterial genera or species in different seasons (see Table 5).

### Concentration of the coarse fraction of bacteria versus coarse solid particles

**Fig. 3:**
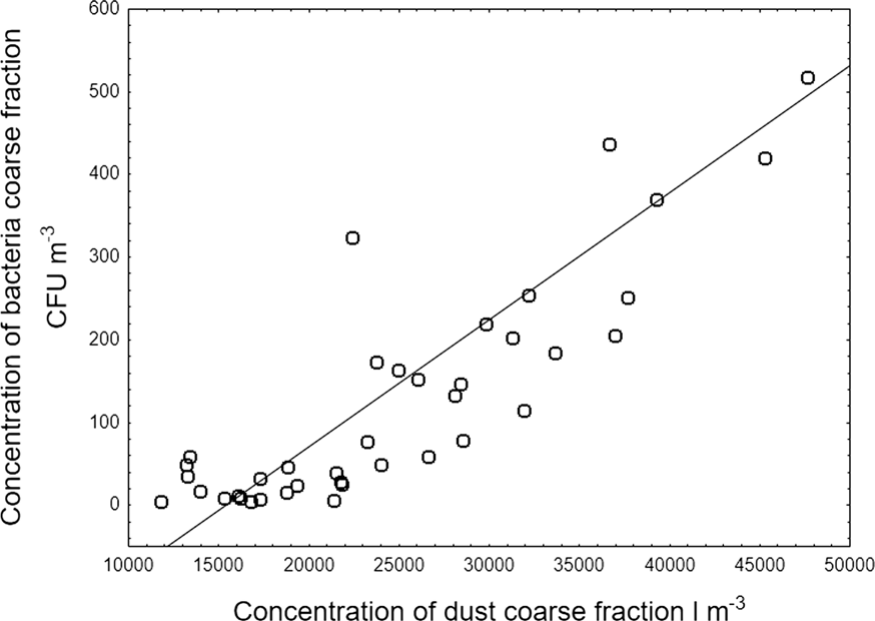
Concentration of the coarse fraction of bacteria versus coarse solid particles (suspended dust) in the atmospheric air in Gliwice

Figure 3 shows the relationship between the concentrations of the coarse fraction of airborne bacteria and suspended dust (solid particles) obtained in Gliwice, averaged for two seasons (spring and winter). We observed very frequently the days when the concentration of solid particles was about 15,000–20,000 coarse particles per cubic metre but the only limited number of days when the concentration exceeded 35,000 coarse particles per cubic metre and almost all of these days were in spring or in winter, i.e. in the heating season. In Poland, the heating process is based on the coal combustions what generates the huge emission of particles, including coarse particles. Therefore, we selected only spring and winter days for this analysis.

We assumed that the coarse mode means that solid and biological particles have an aerodynamic diameter *d*_ae_ > 3.3 µm. It can be seen from Fig. 3 that at the significant level of 0.05, there is a strong correlation between the concentration of coarse bacteria and dust, equal to 0.74. The equation of the regression line for this graph can be assumed as follows: *y* = − 237.16 + 0.0154·*x*.

The question of whether such a result could be expected is open to debate. In fact, recently, Haas et al. (2013) found in Graz, Austria, that the concentrations of mesophilic bacteria correlated with all particle fractions, especially that of coarse particles. On the other hand, Chi and Li (2007) obtained only weak correlations between bioaerosol concentrations and air pollutants, including particulate matter.

It can be concluded that the increase in the concentration of coarse airborne solid particles (dust) stimulates the increase in the contribution of coarse bacterial particles in the total concentration of bacterial aerosol. This may be due to the adhesion of small, respirable bacteria (less than 3.3 μm) to the coarse dust particles suspended in the air.

**Fig. 4:**
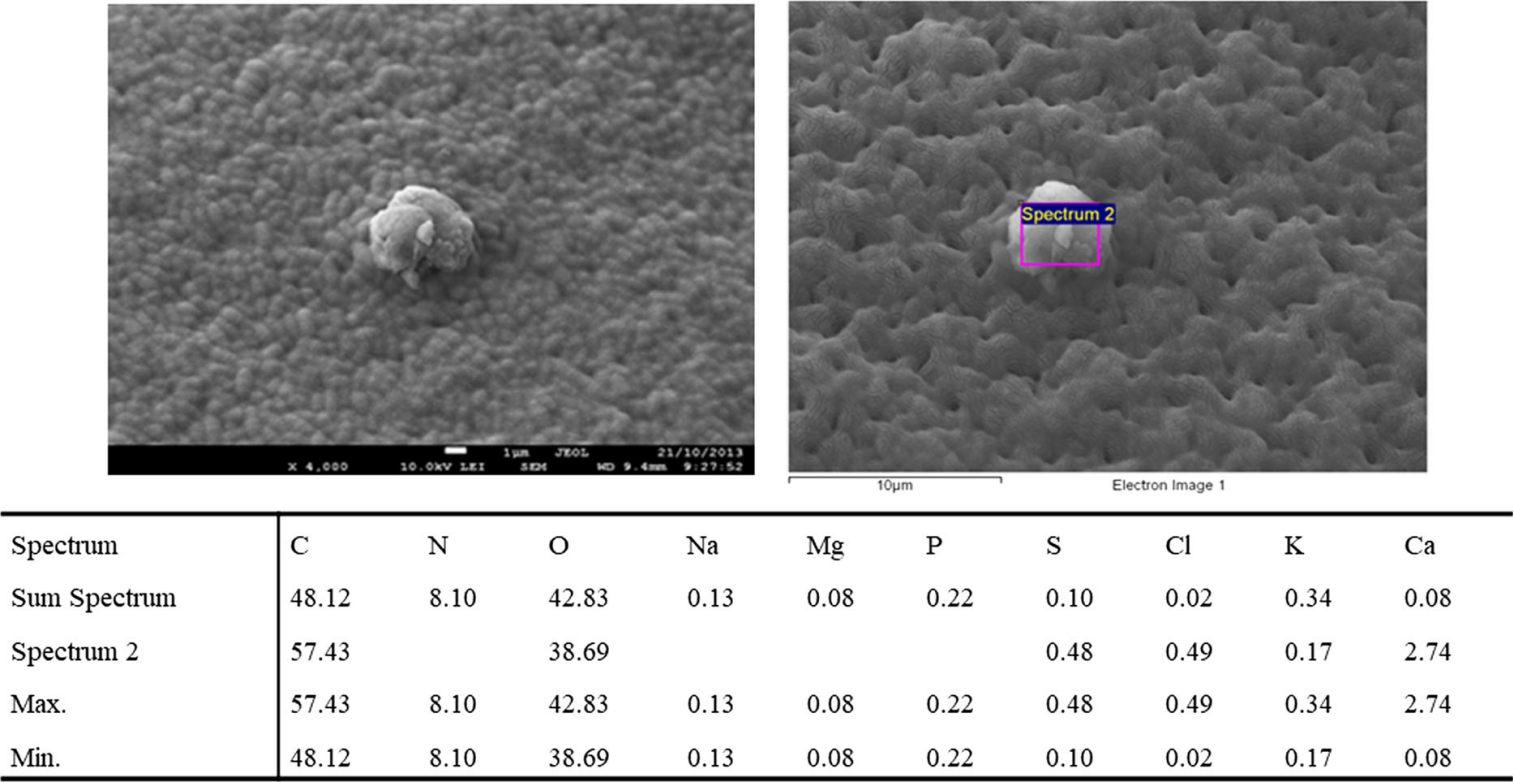
Micrograph of part of a bacterial colony incubated on TSA agar with the results of the chemical analysis of the solid particles found inside the bacterial colonies after sampling during the second stage of the Andersen impactor (cut-off diameter: 4.7–7 µm). Sample 1 was collected in the atmospheric air in Gliwice

**Fig. 5:**
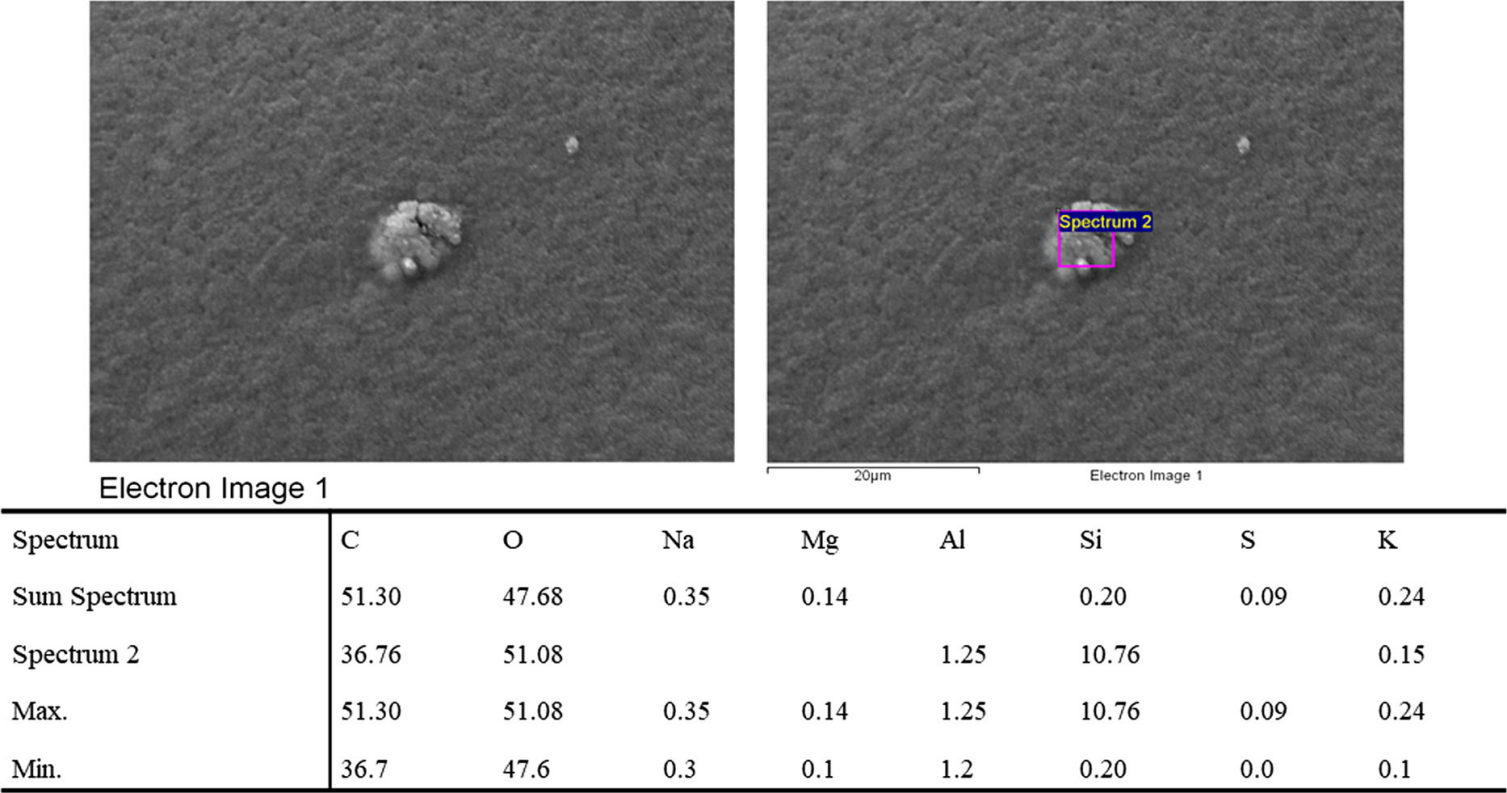
Micrograph of part of a bacterial colony incubated on TSA agar with the results of the chemical analysis of the solid particles found inside the bacterial colonies after sampling during the second stage of the Andersen impactor (cut-off diameter: 4.7–7 µm). Sample 2 was collected in the atmospheric air in Gliwice

The hypothesis that the culturable bacteria are attached to particulate matter in the atmospheric air in Gliwice, especially in winter, seems to be supported by the analysis of Figs. 4 and 5. These pictures (selected as examples) were made with the scanning electron microscope and show solid particles located on the colony of airborne bacteria collected from the atmospheric air in Gliwice during stage two (cut-off diameter > 4.7–7 µm). It is possible that one or more small bacterial cells attached to this coarse solid particle and formed this agglomerate. During the sampling process, this agglomerate was classified by the impactor as one big bacterial particle. The tables below these pictures contain the results of the chemical analysis of the solid particles found inside the bacterial colonies. It was found that these particles contained calcium chloride, calcium carbonates and aluminosilicates.

The adhesion of fine airborne bacteria to the coarse dust particles, if true, might be a very important phenomenon, because a number of originally fine bacteria after inhalation would deposit in the upper instead of the lower part of the respiratory system. However, the hypothesis that the linear increase in the concentration of coarse bacteria with increasing concentrations of coarse solid particles (dust) is caused only by meteorological factors cannot be excluded. Certainly, this phenomenon needs future study.

### Analysis of the meteorological factors

Meteorological conditions, including temperature, wind velocity and relative humidity, are among the most important factors influencing the concentrations of outdoor bacteria (Di Giorgio et al. 1996; Jones and Harrison 2004; Lighthart et al. 2009; Mouli et al. 2005; Wu et al. 2012). The Spearman correlation coefficients between bacterial aerosol concentrations in every season and meteorological parameters are presented in Table 4.

**Table 4:** Spearman correlation coefficients between bacterial aerosol concentrations against meteorological factors

	Bacteria	T (°C)	RH (%)	W (km/h)	UV (W/m^2^)
Spring					
Bacteria	1				
T (°C)	− *0.284*	1			
RH (%)	0.129	− *0.509*	1		
W (km/h)	− 0.012	− 0.211	− 0.109	1	
UV (W/m^2^)	− *0.349*	*0.648*	− *0.532*	− 0.048	1
Summer					
Bacteria	1				
T (°C)	− *0.687*	1			
RH (%)	0.222	− 0.225	1		
W (km/h)	*0.266*	− 0.202	0.134	1	
UV (W/m^2^)	− *0.385*	*0.456*	− *0.522*	− *0.226*	1
Autumn					
Bacteria	1				
T (°C)	*0.499*	1			
RH (%)	0.082	− *0.258*	1		
W (km/h)	− *0.282*	0.090	− 0.173	1	
UV (W/m^2^)	− *0.407*	− *0.602*	− *0.635*	− 0.117	1
Winter					
Bacteria	1				
T (°C)	*0.762*	1			
RH (%)	*0.476*	*0.663*	1		
W (km/h)	− 0.087	− 0.020	− 0.230	1	
UV (W/m^2^)	− *0.488*	− *0.631*	− *0.607*	0.054	1

Italic entries indicate that the correlation is significant at the 0.05 level; temperature, T (°C); relative humidity, RH (%); wind velocity, W (km/h); UV radiation, UV (W/m^2^)

#### Influence of air temperature and UV radiation

As listed in Table 4, there is a significant positive correlation between temperature and bacterial bioaerosol concentrations in autumn and winter, but there is a negative correlation in summer and spring. The Spearman correlation coefficient was 0.499, 0.762 in autumn and winter, respectively, and − 0.687, − 0.284 in summer and spring, respectively (*p* < 0.05). During autumn and winter, the low temperatures decrease cell membrane fluidity and slow down microbial activity; the increased atmospheric temperature promotes the growth and release of bacteria—thus, a positive correlation is observed. On the other hand, in summer and spring, the temperature is higher and is related to strong ultraviolet radiation. This is confirmed by a significant positive Spearman correlation coefficient of 0.648 and 0.456 in spring and summer, respectively (*p* < 0.05), meaning that these conditions are not suitable for the propagation and growth of bacteria and lead to the denaturation and inactivation of proteins (Zhong et al. 2016).

Other studies (Di Giorgio et al. 1996; Liu et al. 2015; Mouli et al. 2005; Zhong et al. 2016) indicated that the microbial concentration in the atmosphere was significantly positively correlated with the atmospheric temperature. However, in the summer, when the highest values of temperature were recorded, we found a decrease in bacterial aerosol concentrations. The reason for this decline may be the relatively high values of temperature, and primarily, the strong UV radiation of the sun noted in this season. This result accords well with the available literature data. Many authors suggested that high temperature with a strong UV intensity in summer may reduce the outdoor bacterial levels (Chi and Li 2007; Fang et al. 2007; Wang et al. 2010; Wu et al. 2012).

During our study, UV radiation was significantly different for each season. Table 4 shows the correlation between bacterial aerosol concentrations and UV radiation. During all seasons, we observed a statistically significant negative relationship between bacterial aerosol and UV radiation, with values of − 0.349, − 0.385, − 0.407 and − 0.488 (*p* < 0.05), for spring, summer, autumn and winter, respectively. Therefore, airborne bacteria in Gliwice would be more likely to propagate and grow at relatively low UV radiation levels, whereas a comparatively high UV radiation value would inhibit their growth.

#### Influence of relative humidity

High water activity is typically favourable for bacterial growth because the bacteria can absorb this water from their living substrates for metabolism. In addition, high relative humidity may result in the clumping of the cells, which possibly increases odds of cell survival (Kallawicha et al. 2015). The microbial activity of bioaerosols will be inhibited if the RH is too low because a dry environment depresses the metabolism and physiological activities of micro-organisms (Zhong et al. 2016).

In our study, a positive correlation between RH and bacterial levels was found. As indicated in Table 4, the correlation was higher and only statistically significant in winter (*p* < 0.05). According to Jones and Harrison (2004), moisture in the air could alter the integrity of cell walls and viral coats, and thus, increased air humidity could promote the growth and release of bacteria.

#### Influence of wind velocity

Wind may resuspend bacteria from soil or plant surfaces (Jones and Harrison 2004; Lighthart and Stetzenbach 1994). According to the general resuspension phenomenon, higher wind speed may decrease ambient bacterial levels by atmospheric dilution (Sabariego et al. 2000).

In our study, the Spearman correlation coefficient was − 0.012 and − 0.087 (*p* > 0.05) in spring and winter, respectively. Therefore, there was no relationship between wind velocity and bacterial concentrations in these seasons. This result is in a good agreement with the observations of Li et al. (2017). The correlation coefficient for autumn was − 0.282, and for summer it was 0.266 (*p* < 0.05). To find, however, the more precisely relationship between the level of airborne bacteria and wind speed, the huge data should be analysed. In particular, it is important to measure the concentration of bacterial aerosol for the velocity of wind ranged from less than 1 km h^−1^ to more than 20 km h^−1^.

### Identification of bacterial aerosol

Five groups of culturable bacteria were identified: gram-positive rods that form endospores, non-sporing gram-positive rods, gram-positive cocci, *actinomycetes* and gram-negative rods (Table 5). The dominant group of culturable bacteria isolated from the atmospheric air in Gliwice in every season was that of gram-positive rods that form endospores (spring 37%, summer 62%, autumn 53%, winter 52%). The largest share of this group of bacteria was observed in the summer, and this may be related to the fact that this group of bacteria has a greater resistance to UV radiation and desiccation from a reduction in relative humidity because of their spore form (Aydogdu et al. 2010; Mahdy and El-Sehrawi 1997). The spore-forming bacteria represent the largest species present in the atmosphere, a very harsh environment for bacteria (Shaffer 1997). The second group of bacteria commonly found in the atmospheric air in Gliwice were gram-positive cocci (spring 23%, summer 26%, autumn 30%, winter 33%).

**Table 5:** Culturable bacterial genera/species identified outdoors during every season

Bacteria	Spring	Summer	Autumn	Winter
Percentage of genera/species in total bacteria concentration (%)				
Gram-positive cocci, including	**23**	**26**	**30**	**33**
*Micrococcus* sp.	17	14	17	14
*Staphylococcus sciuri*	3	4	10	10
*Staphylococcus lentus*	2	7	n.i.	9
*Kocuria rosea*	1	1	3	n.i.
Non-sporing gram-positive rods, including	**26**	10	**11**	**10**
*Arthrobacter* sp.	n.i.	n.i.	2	n.i.
*Brevibacterium* sp.	15	10	3	10
*Corynebacterium auris*	11	n.i.	6	n.i.
Sporing gram-positive rods, family *Bacillaceae*, including	**37**	**62**	**53**	**52**
*Bacillus cereus*	19	23	12	18
*Bacillus pumilus*	14	16	10	15
*Bacillus subtilis*	n.i.	19	18	16
*Bacillus circulans*	4	4	13	3
Actinomycetes	**13**	**1**	**4**	**2**
*Rhodococcus* sp.	4	1	2	n.i.
*Streptomyces* sp.	9	n.i.	2	2
Gram-negative rods, including	**1**	**1**	**2**	**3**
*Pseudomonas* sp.	1	1	2	3

*n.i.* not identify

**Fig. 6:**
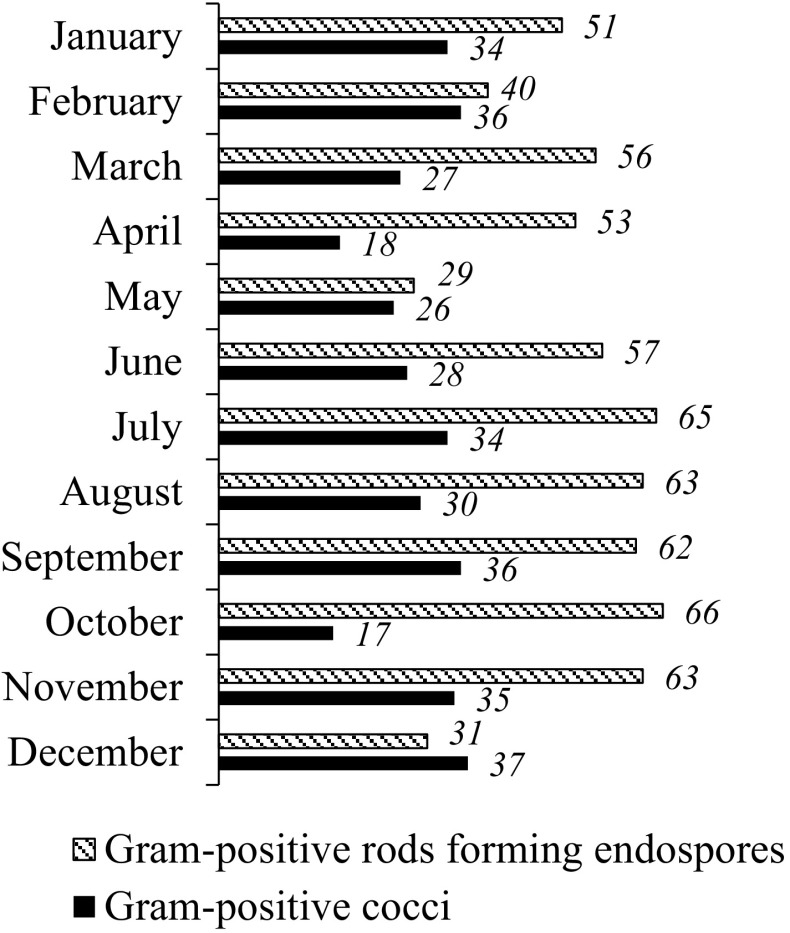
Percentages of the two most isolated groups of bacteria from the outdoor air of Gliwice (gram-positive rods that form endospores and gram-positive cocci)

During each month, the most commonly isolated bacterial group was that of gram-positive rods that form endospores, in which *Bacillus* was the most isolated genus. The second most frequently isolated group of bacteria was that of gram-positive cocci, in which the most isolated genus was *Micrococcus* (Fig. 6).

Similar results were obtained in atmospheric air in Ciechocinek, a health resort in Poland, where the *Bacillus* was the most common among the isolated bacteria and *Micrococcus* was also abundant (Burkowska et al. 2012). The study carried out by Aydogdu et al. (2010) in outdoor air in Turkey also shows that the predominant airborne bacteria were gram-positive rods that form endospores and that *Bacillus* was the most isolated genus. Bartlett et al. (2004) similarly found that the dominant genera in the outdoor air in Canada were *Bacillus* and *Micrococcus.* Fang et al. (2007) also showed that *Bacillus* and *Micrococcus* were the most abundant among 30 genera in the atmospheric air in Beijing.

The statistical analysis of bacterial identification in outdoor air points to significant differences for gram-positive rods that form endospores between May and 6 months (from June to November) and between December and 6 months (from June to November). For gram-positive cocci, we found significant statistical differences between May and 2 months October and November. The difference for gram-positive cocci was also statistically different between November and April, May, June (*p* < 0.05).

Gram-positive rods are commonly found in soil and water habitats, and many of them are part of the normal skin and mucous membrane flora of humans and various animals. The virulence of gram-positive rods is highly varied. Many of them have the potential to be opportunistic pathogens, capable of producing disease only in persons with compromised host resistance (Burkowska et al. 2012). In the present study, airborne gram-positive bacteria were the most abundant in each season. They accounted for almost 90% while the gram-negative bacteria were present in less than 5% of the outdoor samples. This result is in agreement with other reports (Aydogdu et al. 2010; Shaffer 1997; Bartlett et al. 2004; Brągoszewska et al. 2016).

## Conclusions

The average concentration of the total bacterial aerosol collected in the outdoor air in Gliwice, over four seasons (spring, summer, autumn and winter) differed significantly. The average levels of bacterial aerosol concentrations in the outdoor air ranged from approximately 50 to 300 CFU m^−3^. The highest concentrations of bacterial aerosol were recorded during the spring season (306 CFU m^−3^), while the lowest were observed in the winter (49 CFU m^−3^). The average size of culturable bacterial aerosol outdoors over the study period was 199 CFU m^−3^. Comparing these results with other data, obtained, for example, in Poznań, Poland (Bugajny et al. 2005) or in Beijing (Fang et al. 2008), it can be concluded that the atmospheric air of Gliwice, in terms of microbiological pollution, is relatively clean.

We analysed the correlation between bacterial aerosol concentrations and meteorological parameters. Statistically, the most important meteorological factors in the viability of airborne bacteria were temperature (T) and UV radiation (UV). In spring, the Spearman correlation coefficients for T or UV with bacterial aerosol concentrations were − 0.284 and − 0.349 (*p* < 0.05), in summer − 0.687 and − 0.385 (*p* < 0.05), in autumn 0.499 and − 0.407 (*p* < 0.05), in winter 0.762 and − 0.488 (*p* < 0.05). The influence of UV radiation was negative during each season. Low UV radiation led to higher airborne bacteria concentrations, while high UV contributed to lower bacterial aerosol concentrations. However, the temperature had a different impact in each of the seasons. During autumn and winter, a strong positive correlation was observed between temperature and bacterial aerosol concentrations. These concentrations were high in relatively warm temperatures and were low in cooler temperatures. During spring and summer, the opposite was observed. The higher temperature decreased the ability of ambient bacteria to survive. This dependence was caused by the significant correlation between temperature and UV radiation. The Spearman correlation coefficients between T and UV were of 0.648 and 0.456 (*p* < 0.05) in spring and summer, respectively, and − 0.602 and − 0.631 (*p* < 0.05) in autumn and winter, respectively. Relative humidity was positively correlated with bacterial aerosol concentrations; however, this relationship was only statistically significant in winter (0.476; *p* < 0.05). Wind velocity was found to have no statistically significant correlation with bacterial aerosol concentrations in spring and winter; however, in the summer and autumn, statistically significant correlation was found.

Concentration of coarse bacterial particles was highly correlated with the concentration of coarse particles of atmospheric particulate matter, which could suggest the rafting of fine bacterial cells on the surface of coarse solid particles.

The size distributions obtained in four seasons were unimodal, with a peak found in the range of bacterial diameters from 3.3 to 4.7 µm. In the cooler seasons, the particle size distribution was more “flattened”, but future studies are needed to find a clear reason for this result.

In each season, the largest group of airborne bacteria were gram-positive rods that form endospores, while the second group, commonly isolated, were gram-positive cocci. The dominant airborne bacteria species detected in the outdoor air were *Bacillus cereus*, *Bacillus subtilis, Bacillus pumilus* and *Micrococcus* sp.

The results obtained may serve as a reference for future assessments and provide to be a useful contribution to (a) reviews of policies, (b) implementation of public health prevention programmes and (c) the formulation of recommendations aimed at providing healthier outdoor environments.
